# Children on the autism spectrum update their behaviour in response to a volatile environment

**DOI:** 10.1111/desc.12435

**Published:** 2016-08-06

**Authors:** Catherine Manning, James Kilner, Louise Neil, Themelis Karaminis, Elizabeth Pellicano

**Affiliations:** ^1^ Department of Experimental Psychology University of Oxford UK; ^2^ Wellcome Trust Centre for Neuroimaging UCL Institute of Neurology University College London UK; ^3^ Centre for Research in Autism and Education (CRAE) UCL Institute of Education University College London UK; ^4^ School of Psychology University of Western Australia Australia

## Abstract

Typical adults can track reward probabilities across trials to estimate the volatility of the environment and use this information to modify their learning rate (Behrens *et al*., 2007). In a stable environment, it is advantageous to take account of outcomes over many trials, whereas in a volatile environment, recent experience should be more strongly weighted than distant experience. Recent predictive coding accounts of autism propose that autistic individuals will demonstrate atypical updating of their behaviour in response to the statistics of the reward environment. To rigorously test this hypothesis, we administered a developmentally appropriate version of Behrens *et al*.'s (2007) task to 34 cognitively able children on the autism spectrum aged between 6 and 14 years, 32 age‐ and ability‐matched typically developing children and 19 typical adults. Participants were required to choose between a green and a blue pirate chest, each associated with a randomly determined reward value between 0 and 100 points, with a combined total of 100 points. On each trial, the reward was given for one stimulus only. In the *stable* condition, the ratio of the blue or green response being rewarded was fixed at 75:25. In the *volatile* condition, the ratio alternated between 80:20 and 20:80 every 20 trials. We estimated the learning rate for each participant by fitting a delta rule model and compared this rate across conditions and groups. All groups increased their learning rate in the volatile condition compared to the stable condition. Unexpectedly, there was no effect of group and no interaction between group and condition. Thus, autistic children used information about the statistics of the reward environment to guide their decisions to a similar extent as typically developing children and adults. These results help constrain predictive coding accounts of autism by demonstrating that autism is not characterized by uniform differences in the weighting of prediction error.

## Research highlights

- Predictive coding models have recently been proposed to account for the complex autism phenotype.
- Here, we test a key prediction from predictive coding accounts using a probabilistic learning task.
- Autistic children did not have generally elevated learning rates compared to typically developing children, and updated their learning rate in a volatile reward environment.
- These results suggest that autism is not characterized by uniformly high and inflexible weighting of prediction errors.

## Introduction

The decisions we make in a given moment are informed by expectations derived from the outcomes of similar decisions we have made in the past (Behrens, Woolrich, Walton & Rushworth, 2007; Louie & Glimcher, 2012; Summerfield & Tsetsos, 2015). Rather than assigning equal weight to all previous outcomes, neurotypical adults can track the statistics of the environment in order to determine how much weight should be given to new information. When the reward environment is stable, people take account of previous outcomes over many trials to guide their decisions (Behrens *et al*., 2007; O'Reilly, 2013). Yet, when the reward environment is volatile (i.e. fluctuating over blocks of trials), people weight their recent experience more strongly than their distant experience (Behrens *et al*., 2007). The relative weighting given to recent and distant trials is reflected in a person's learning rate (Dayan, Kakade & Montague, 2000). In a stable environment, neurotypical adults demonstrate a low learning rate (Behrens *et al*., 2007), as the history of outcomes is more predictive of the current state of the environment than the outcomes in the most recent trials. In a volatile environment, adults demonstrate an increased learning rate, whereby they ‘take notice’ of the outcomes of more recent trials and use these to modify their behaviour (Behrens *et al*., 2007).

The ability to take account of previous information has been the focus of recent advances in theories of autistic perception and cognition (see Brock, 2014, for review). Applying a Bayesian framework, Pellicano and Burr (2012a) suggested that autistic^1^ individuals make less use of prior information than typical individuals. Under the Bayesian framework, the percept (or posterior) results from a combination of incoming sensory information (the likelihood) and previous information (priors), the weighting of which depends on their respective precision. Pellicano and Burr suggested that autistic individuals have attenuated (broader) priors, meaning that their perception is more dominated by the incoming sensory information. While the theory initially focused on perception, the weaker influence of prior information could potentially account for a range of aspects of the autism phenotype, such as social functioning (Pellicano & Burr, 2012b).

Elaborations of this account have used the predictive coding framework, which provides a biologically plausible implementation of Bayesian inference (see Clark, 2013, for review), replacing priors and sensory evidence with predictions and prediction errors, respectively. According to the predictive coding approach, the brain aims to predict what will happen next and attempts to minimize prediction error – the discrepancy between the prediction and reality. Predictions emerging from higher brain areas are used to attempt to ‘explain away’ the input from lower brain areas and prediction errors are passed up the hierarchy to inform higher‐level expectations. The influence of prior beliefs relative to sensory evidence is controlled by the precision assigned to prediction errors at each level of the hierarchy (Friston, 2008). This balance may be atypical in autistic individuals, who may have a low precision of prior information relative to that of the sensory information (Friston, Lawson & Frith, 2013; Lawson, Rees & Friston, 2014). Van de Cruys, Evers, Van der Hallen, Van Eylen, Boets *et al*. (2014) made a more specific proposal that the precision of prediction errors is uniformly high and inflexible in individuals with autism. Finally, Sinha, Kjelgaard, Gandhi, Tsourides, Cardinaux *et al*. (2014) proposed that autistic individuals have impairments in estimating the conditional probability of future events or states given a previous state occurring, particularly when the relationship between events is probabilistically weak and/or when events are separated by long temporal intervals. Yet, autistic individuals may excel at learning rules, due to heightened learning of novel stimuli (Sinha *et al*., 2014).

Atypical deployment of previous experience has been suggested to relate to a range of autism symptoms, such as insistence on sameness, repetitive behaviours, and impaired social functioning (Lawson *et al*., 2014; Van de Cruys *et al*., 2014). Yet, experimental studies are required to rigorously test the mechanisms proposed by such approaches. If autistic individuals do not use previous experience in the same way as typical individuals – either through weakened priors or atypical predictive mechanisms – they may behave differently in a task that involves tracking the statistics of the reward environment, such as that used by Behrens *et al*. (2007). No previous studies have assessed whether children on the autism spectrum increase their learning rate in response to environmental volatility. Yet, reversal learning studies give some insight into how individuals with autism deal with probabilistic information. Most of these studies suggest that individuals with autism can learn initial reinforcement probabilities, but demonstrate difficulties in switching when these probabilities reverse (e.g. Solomon, Smith, Frank, Ly & Carter, 2011; South, Newton & Chamberlain, 2012) or in maintaining new response probabilities (D'Cruz, Ragozzino, Mosconi, Shrestha, Cook *et al*., 2013). Such difficulties in reversal learning may reflect executive functioning difficulties in autism (Pennington & Ozonoff, 1996; see Pellicano, 2012, for review). Yet, these studies do not address how higher‐order statistics about the environment – such as volatility – affect autistic individuals’ responses.

Robic, Sonié, Fonlupt, Henaff, Touil *et al*. (2015) presented a cued decision‐making task to 14 autistic adults and 15 neurotypical adults whilst manipulating the volatility of the environment (stable, unstable). A social cue (video of a human actor) or non‐social cue (arrow) was presented before participants chose between one of two options, with both the reliability of the cue and the reward probabilities of each option being manipulated throughout the experiment. The authors recorded the proportion of correct choices made by participants, and reported that fewer individuals with autism met a criterion of 60% correct than typical individuals when reward probabilities fluctuated (unstable condition), but not when reward probabilities were fixed (stable condition), and that individuals with autism performed particularly poorly in response to the social cue. The authors concluded that individuals with autism have particular difficulties in learning reward probabilities when the reward environment is unstable and involves a social aspect. Yet, importantly, half of the participants with autism did meet a criterion of 60% in the unstable condition, suggesting considerable individual differences in this performance metric. In this study, we used a more fine‐grained measure to characterize behavioural responses to environmental volatility in autism.

We administered a child‐friendly version of Behrens *et al*.'s (2007) task to 34 cognitively able autistic children, 32 typically developing children and 19 typical adults. Participants were required to choose between two stimuli under two conditions: in the *stable* condition, the probability of each stimulus being rewarded was fixed. In the *volatile* condition, the probability of each stimulus being rewarded alternated every 20 trials. A crucial difference between this paradigm and those used in previous reversal learning studies in autism (e.g. D'Cruz *et al*., 2013; Solomon *et al*., 2011; South *et al*., 2012; Robic *et al*., 2015) was that each stimulus was associated with a reward value that varied trial by trial. This paradigm allowed us to model the learning rates of participants (cf. Behrens *et al*., 2007), and in turn to address specific predictions derived from Bayesian and predictive coding accounts of autism.

Pellicano and Burr (2012a) suggested that individuals with autism are less influenced by information presented in the past, due to reduced priors. Van de Cruys *et al*. (2014) suggested that individuals with autism have very precise prediction errors, which would mean that they should heavily weight violations to their expectations and update their behaviour accordingly. Sinha *et al*. (2014) also predicted heightened learning to novel stimuli. Thus, according to these three accounts, children on the autism spectrum may demonstrate a generally elevated learning rate, emphasizing the outcomes of more recent trials (and thus, violations to their predictions) more than typical children. Van de Cruys *et al*. also made a further specific proposal that individuals with autism do not flexibly weight their prediction errors, which may mean that they do not modify their learning rate in the volatile condition compared to the stable condition to the same extent as typical individuals.

Alongside these primary hypotheses, we were also interested in examining possible relationships between task performance and anxiety. Anxiety commonly co‐occurs with autism (White, Oswald, Ollendick & Scahill, 2009), and high levels of trait anxiety have been linked to *reduced* updating of behaviour in response to volatility in an aversive version of Behrens *et al*.'s task (Browning, Behrens, Jocham, O'Reilly & Bishop, 2015). This link is particularly interesting given a suggestion that atypical predictive mechanisms may give rise to increased anxiety in autistic individuals (Sinha *et al*., 2014). To this end, we investigated relationships between behaviour updating and parent‐reported anxiety in our children with and without autism.

## Materials and methods

### Participants

Three groups of participants were tested: 34 autistic children, 32 typically developing children and 19 typical adults (see Table 1 for participant characteristics). The autistic children had previously received an independent clinical diagnosis of an autism spectrum condition according to ICD‐10 (World Health Organization, 1993) or DSM‐IV (American Psychiatric Association, 2000) criteria. Typically developing children had no parent‐reported diagnoses of developmental conditions and typical adults reported no previous diagnoses of developmental conditions. Children's parents completed the Social Communication Questionnaire (SCQ; Rutter, Bailey & Lord, 2003) and children with autism were administered the Autism Diagnostic Observation Schedule (ADOS‐G or ADOS‐2; Lord, Rutter, DiLavore & Risi, 1999; Lord, Rutter, DiLavore, Risi, Gotham *et al*., 2012) using the revised algorithm (Gotham, Risi, Pickles & Lord, 2007; Gotham, Risi, Dawson, Tager‐Flusberg, Joseph *et al*., 2008). All autistic children scored above threshold for an autism spectrum condition on one or both of these measures and all typically developing children scored below the cut‐off for autism on the SCQ (< 15; Rutter *et al*., 2003) (see Table 1 for scores).

**Table 1:** Participant characteristics

	Children with autism	Typically developing children	Typical adults
*N*	34	32	19
Gender (*n* males: *n* females)	29: 5	22: 10	7: 12
Age (years; months)			
Mean (*SD*)	9;11 (2;0)	9;2 (1;10)	24;2 (3;9)
Range	7;0–14;3	6;6–13;2	18;5–33;1
Performance IQ			
Mean (*SD*)	105.44 (14;94)	104.84 (14.11)	
Range	79–141	78–131	
Verbal IQ			
Mean (*SD*)	100.15 (17.47)	108.03 (11.04)	
Range	71–130	86–132	
Full‐scale IQ			
Mean (*SD*)	102.94 (15.46)	107.28 (10.66)	
Range	76–129	89–131	
SCQ score			
Mean (*SD*)	23.78 (7.35)	5.77 (4.04)	
Range	5–35	1–14	
Spence Children's Anxiety			
Mean (*SD*)	33.52 (20.35)	19.78 (10.67)	
Range	6–76	6–43	
ADOS total score			
Mean (*SD*)	10.45 (4.64)		
Range	2–21		

Notes

SCQ = Social Communication Questionnaire (Rutter *et al*., 2003). ADOS = Autism Diagnostic Observation Schedule (Lord *et al*., 1999, 2012). Verbal, Performance and Full‐Scale IQ scores were derived from the Wechsler Abbreviated Scales of Intelligence (WASI‐II; Wechsler, 2011).

The groups of autistic and typical children were matched in terms of age, *t*(64) = 1.65, *p *=* *.10, performance IQ, *t*(64) = .17, *p *=* *.87 and full‐scale IQ, *t*(58.78) = 1.33, *p *=* *.19, as assessed by the Wechsler Abbreviated Scales of Intelligence, Second Edition (WASI‐II, Wechsler, 2011). The children with autism had lower verbal IQ scores than typical children, consistent with their clinical profile, *t*(56.17) = 2.21, *p *=* *.03. Anxiety was measured using the parent‐report version of the Spence Children's Anxiety Scale (SCAS‐P; Nauta, Scholing, Rapee, Abbott, Spence *et al*., 2004), which was returned by 58 parents. The children with autism (*n *=* *31) had significantly higher scores than typical children (*n *=* *27), *t*(46.58) = 3.28, *p *=* *.002.

### Procedure

This study was approved by the UCL Institute of Education's Research Ethics Committee and was conducted in accordance with the principles of the Declaration of Helsinki. Parents and adult participants gave their written informed consent and children provided their verbal assent prior to participation.

We adapted Behrens *et al*.'s task into a child‐friendly, pirate‐themed game. The task began with an initial familiarization phase, designed to introduce the participants to the task, followed by the experimental phase. Both phases were completed in a single session lasting approximately 15 minutes. Adult participants also completed a longer version of the experiment in a separate session lasting approximately 25 minutes (see below). Children and adults were seen individually in a quiet room. The WASI and ADOS were administered in further sessions.

#### Familiarization phase

**Figure 1:**
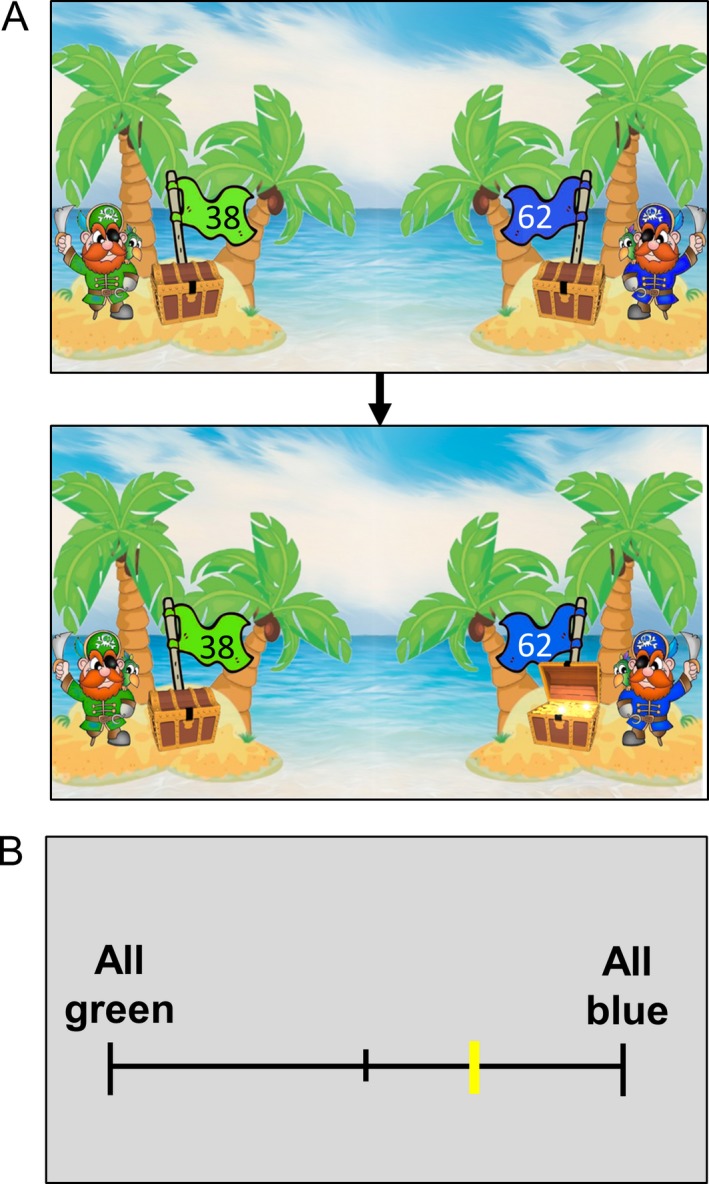
Familiarization phase. (A) Example of a trial in the familiarization phase, where participants passively viewed stimuli. (B) After viewing 20 trials, participants were asked to estimate the ratio in which the green or blue chest contained treasure.

Participants were initially introduced to images on cards showing two pirate chests, one green and one blue, each with a flag displaying a reward value between 0 and 100 points (or ‘coins’), with a combined total of 100 points (Figure 1). The experimenter explained to participants that only one chest would contain treasure on each trial (the rewarded stimulus); the other chest would be empty (the non‐rewarded stimulus). Critically, participants were told that the chest containing treasure could change throughout the game. Next, participants viewed 20 trials in which the chests were opened to reveal which chest was empty and which contained treasure (Figure 1A). The ratio of the blue or green chest being rewarded was fixed at 80:20. Whether the blue or green chest was rewarded most often (i.e. 80% of the time) was randomized across participants. The values on the flags were randomly selected on each trial, with the constraint that the total number of points was always 100. After viewing all 20 trials, participants were asked to estimate the ratio at which the blue and green chests contained treasure, by moving a yellow indicator along a scale ranging from ‘All green’ to ‘All blue’ (Figure 1B). The familiarization phase was repeated for participants who misunderstood the task (two typical children; e.g. those who responded that the treasure was always in the green chest when it was mostly blue, and vice versa).

#### Experimental phase

**Figure 2:**
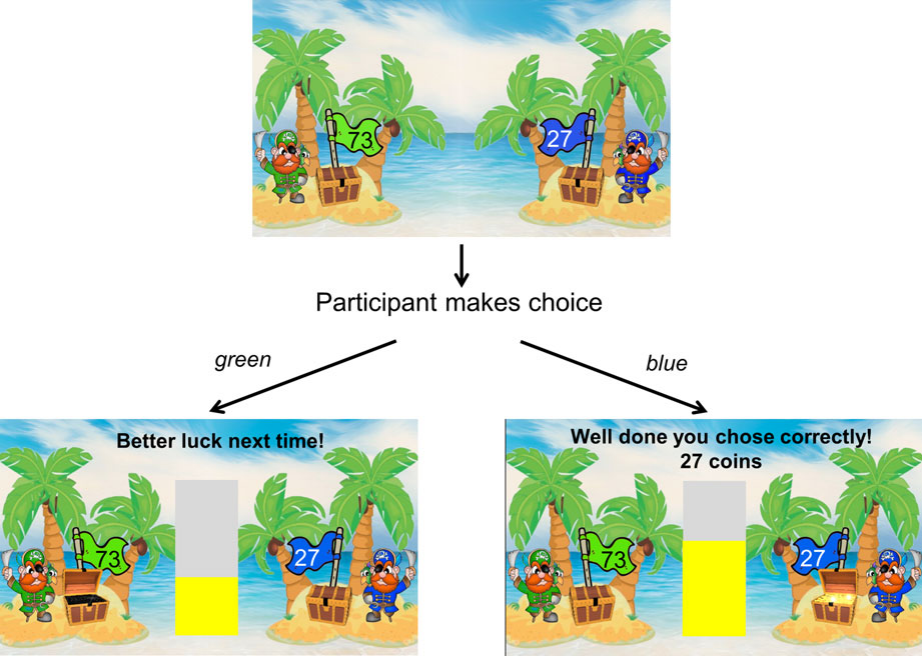
Experimental task. Example of a trial in the test phase in which the rewarded stimulus is the blue pirate chest. If the participant chose the green chest, an empty chest was revealed and no points were awarded. If the participant chose the blue chest, the treasure was revealed and the participant received the number of points (or ‘coins’) displayed on the flag (i.e. 27).

Immediately following the familiarization phase, participants completed the experimental task. As in the familiarization phase, participants were presented with a green and a blue pirate chest on each trial. Here, participants were required to actively choose either the green or blue pirate chest on each trial using response pads (Figure 2). If participants chose the correct (rewarded) stimulus, the chest containing treasure was revealed and they were awarded the number of points (or ‘coins’) indicated on the flag. Participants were given visual feedback (‘Well done, you chose correctly!’) and auditory feedback (the sound of a coin dropping). The points were added to an accumulated total and displayed on a bar chart (Figure 2). If participants chose the incorrect (non‐rewarded) stimulus, an empty chest was revealed and participants did not receive any coins. Visual feedback was provided (‘Better luck next time!’). When participants accumulated enough points to exceed the limits of the bar chart, they were shown a screen that told them they had reached the next ‘level’ (e.g. ‘Congratulations, you are now a level 1 pirate!’). The bar chart was then emptied and participants accumulated points to reach the next level.

During the task, the first 80 trials belonged to the stable condition, where the ratio of the blue or green response being rewarded was fixed at 75:25. The next 80 trials belonged to the volatile condition, whereby the ratio alternated between 80:20 and 20:80 every 20 trials. The conditions followed on from each other without a break and participants were naïve to the reward structure. At the end of the task, the participant's total number of points (or ‘coins’) was displayed, along with the final ‘level’ s/he had reached in the game (e.g. ‘Wow! You reached level 10 and collected 1500 coins!’).

#### Additional experiment for adult participants

The experimental task described above contained fewer trials than that used by Behrens *et al*. (2007) to make it suitable for child participants. To allow direct comparisons with Behrens *et al*.'s original paradigm, adults also completed a longer version of the experiment, which included the same number of trials as presented by Behrens *et al*. The adults completed the longer version in a separate session after the short version, to ensure that the adults’ performance on the short version of the task was directly comparable to that of the children's performance. In this longer version, the stable condition consisted of 120 trials with a fixed ratio of 75:25, and the volatile condition consisted of 170 trials with a ratio that switched between 80:20 and 20:80 every 30 or 40 trials (i.e. 30 – 40 – 30 – 40 – 30). The order of stable and volatile conditions was counterbalanced in this task to confirm that the change in learning rate was not dependent on order of presentation (as originally demonstrated by Behrens *et al*.).

#### Analysis

Prior to testing whether participants modulated their learning rates as a function of volatility, an initial analysis was performed to determine whether the participants’ behaviour could be reliably explained by the ideal Bayesian observer model, and whether this model was the best model to explain the participants’ behaviour in each group. We fitted each individual participant's choices (green or blue) across all trials with a logistic generalized linear model. The design matrix contained a constant term and four different models: (i) ideal observer, (ii) alternating choices, (iii) win‐stay lose‐shift, and (iv) reward value.

The ideal observer regressor was estimated from the sequence of rewarded stimuli across all trials using the model described in Behrens *et al*. (2007). In short, optimal behaviour requires participants to estimate the probability of reward for each stimulus and to compute the expected value as reward probability multiplied by reward size. Given that the amplitude of the reward was random across trials, the ideal observer of the underlying probability of reward success is modelled only by the reward probabilities. In the task, the reward probability varies across trials and is dependent upon the volatility, which changes across the experiment. Therefore, the optimal observer relies on the parameter estimates of the reward probability, the volatility and the confidence in the volatility estimate from the preceding trial, and the latest trial outcome in order to determine decision and learning on the next trial.

The alternating choices regressor tested whether participants simply switched between blue and green options on every consecutive trial. The win‐stay lose‐shift regressor modelled whether the participant's behaviour could be explained by them choosing the same option as the previous trial if that option had been rewarded, and choosing the opposite option if their response on the previous trial had not been rewarded. The reward value regressor was the reward value for the blue option.

The main aim of this study was to test whether the different participant groups modulated their learning rate based on the volatility of the environment. To this end we estimated the learning rate for each participant in the stable and volatile conditions of the task. For the shorter version of the task the learning rate was estimated using the 61 trials from trial 20 to trial 80 in the stable condition, and the 61 trials from trial 90 to trial 150 in the volatile condition. For the longer version of the task, the corresponding windows were the last 81 trials of each of the stable and volatile phases, following Behrens *et al*. (2007). A reinforcement‐learning model was fitted to each participant's decisions in each window. The model has two parts: a ‘predictor’, which estimates the current reward rate given past observations, and a ‘selector’, which generates actions on the basis of these estimates. The predictor is in the form of a simple delta‐learning rule (Rescorla & Wagner, 1972), which has a single free parameter: the learning rate.

## Results

**Figure 3:**
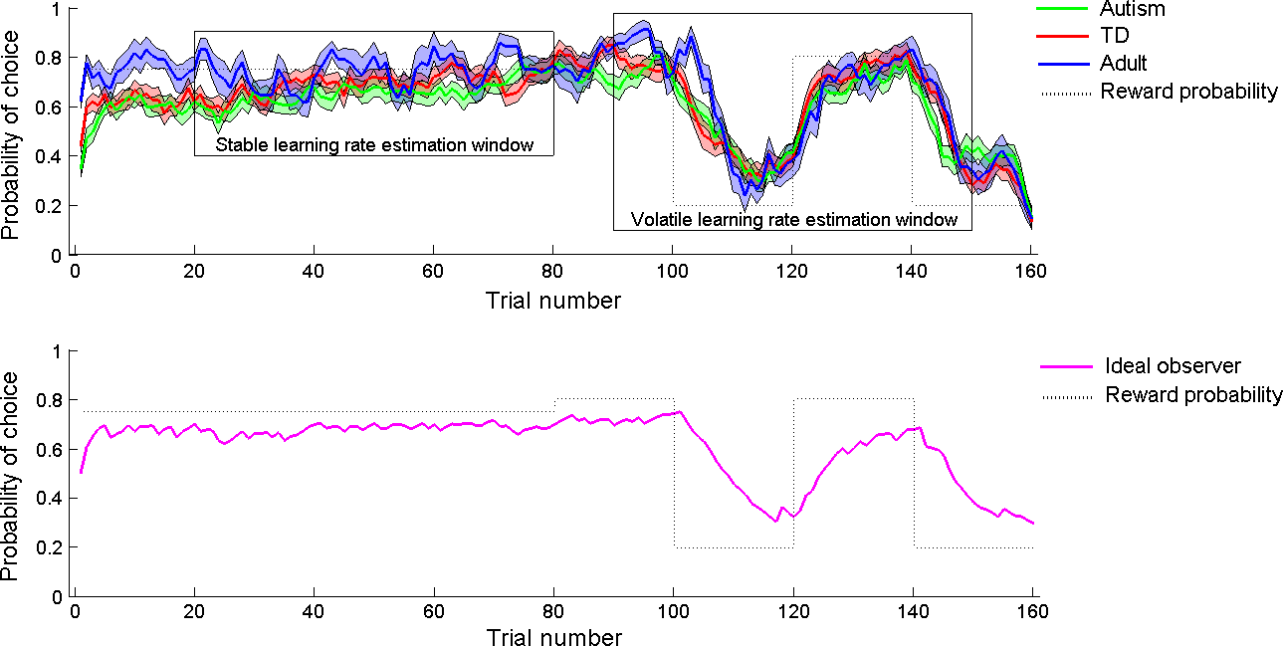
Mean performance of ideal and real observers. The upper panel shows the mean choices ±1 standard error of the mean made by autistic children (green), typically developing (TD) children (red) and adults (blue). The lower panel shows mean ideal observer performance ±1 SEM based on the reward probabilities and values presented to all participants. The dotted lines represent the underlying reward probabilities. Trials 1–80 belong to the stable condition, in which the reward probability is fixed at 75:25. Trials 81–160 belong to the volatile condition, in which the reward probability fluctuates between 80:20 and 20:80 every 20 trials. The probability of choice refers to the probability of choosing the option that was most frequently rewarded in the initial stable condition (which was counterbalanced among participants to be either the blue or green pirate chest). Boxes represent the window of trials over which learning rates were estimated for each individual.

**Figure 4:**
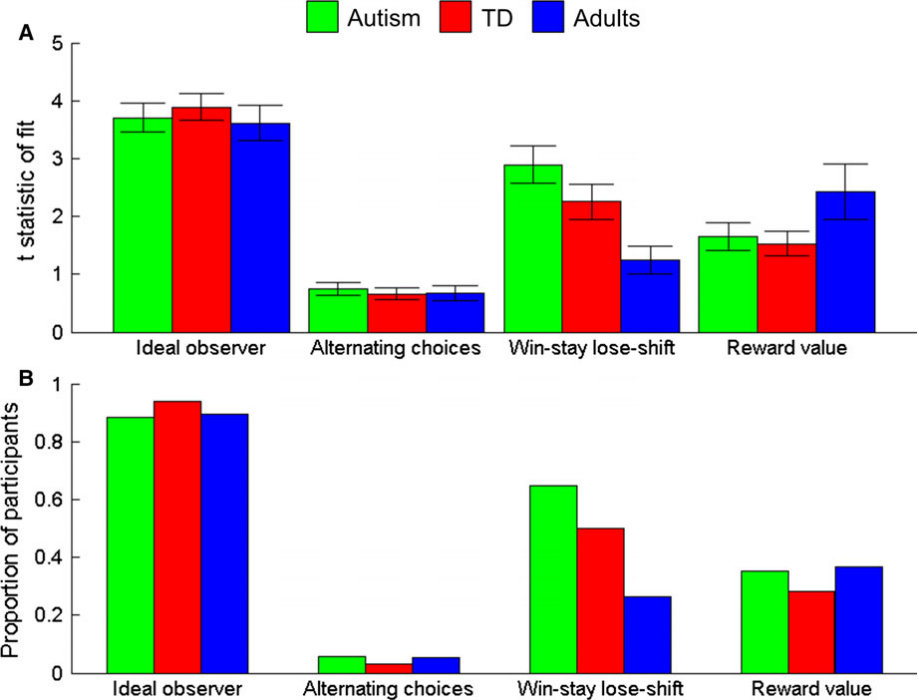
Results of model fitting to participant data. (A) Mean *t*‐statistic of fit for four models to the data of autistic children (green), typically developing (TD) children (red), and typical adults (blue): (1) an ideal observer model, (2) a model which alternates between responses trial‐by‐trial, (3) a win‐stay lose‐shift model (maintaining responses after successes and switching responses after failures), and (4) a model based on the reward value. Error bars represent ±1 standard error of the mean. (B) Proportion of participants in each group whose data were significantly fit with each of the four models (*p *<* *.05).

Figure 3 shows the mean choices made by each group (upper panel) and the ideal observer model (lower panel). The ideal observer model provided the best fit to the data in all groups, compared to the alternating choices, win‐stay lose‐shift and reward value models (Figure 4A). The ideal observer model provided a significant fit (*p *<* *.05) to the choices from the majority of participants in all groups (Figure 4B). We used chi‐squared tests to determine whether the proportion of participants fit by each model varied (i) between child participants and adults, and (ii) between autistic and typically developing children. The only significant difference was that more child participants were fit by the win‐stay lose‐shift model than adults, χ^2^(1) = 5.77, *p *=* *.02. All other comparisons between children and adult participants were non‐significant, *p*s ≥ .68, and there were no significant differences in the proportions of autistic versus typically developing children fit by each model, *p*s ≥ .23.

**Figure 5:**
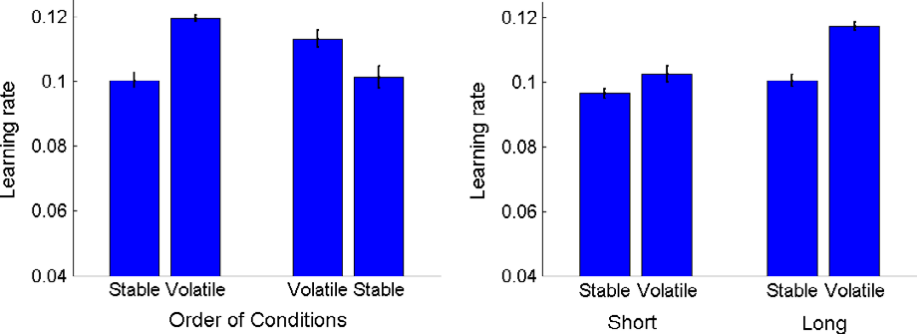
Learning rates of adult observers. Estimated learning rates of adult observers as a function of the order in which stable and volatile conditions were presented (left panel) and the length of the testing session (right panel). Error bars represent ±1 standard error of the mean.

Having demonstrated that the ideal observer model provided the best fit to the data, we next assessed the resulting learning rate estimate. First, we analysed the data from the adult participants on the long version of the experiment (Figure 5) to ensure that we could replicate Behrens *et al*.'s (2007) pattern of results. All learning rates were log‐transformed prior to analysis to meet the assumption of normality required for general linear models.^2^ A mixed‐design ANOVA with condition (stable, volatile) as a within‐participants factor and order of presentation (stable first, volatile first) as a between‐participants factor confirmed that participants increased their learning rate in the volatile condition, *F*(1, 17) = 51.86, *p *<* *.001, ɳ_p_^2^ = .75. In line with Behrens *et al*., the order of presentation did not have a significant effect on learning rate estimates, *F*(1, 17) = .91, *p *=* *.35, and did not interact with condition, *F*(1, 17) = 2.54, *p *=* *.13. Thus, participants had a higher learning rate in the volatile condition than the stable condition even when the volatile condition was presented first.

Next, we compared the adults’ learning rates in the short and long versions of the experiment (Figure 5) using a within‐participants ANOVA with condition (stable, volatile) and length of session (short, long) as factors. Again, we found an overall effect of condition, *F*(1, 18) = 54.81, *p *<* *.001, ɳ_p_^2^ = .75, whereby participants increased their learning rate in the volatile condition. In addition, there was an effect of length, with higher learning rates obtained in the long version of the experiment than the short version, *F*(1, 18) = 24.87, *p *<* *.001, ɳ_p_^2^ = .58. These effects were qualified with an interaction between condition and length, *F*(1, 18) = 19.21, *p *<* *.001, ɳ_p_^2^ = .52. While the increase in learning rate was more pronounced in the long version, post hoc *t*‐tests confirmed that the learning rate increased in both the short, *t*(18) = 3.40, *p *=* *.003, and long version of the experiment, *t*(18) = 8.04, *p *<* *.001. While it is not possible to determine whether the elevated learning rates in the long condition are a result of the increased number of trials, or increased familiarity with the task (as the long condition was presented after the short version), we have established that a clear effect of volatility is present with a reduced number of trials in adult participants.

**Figure 6:**
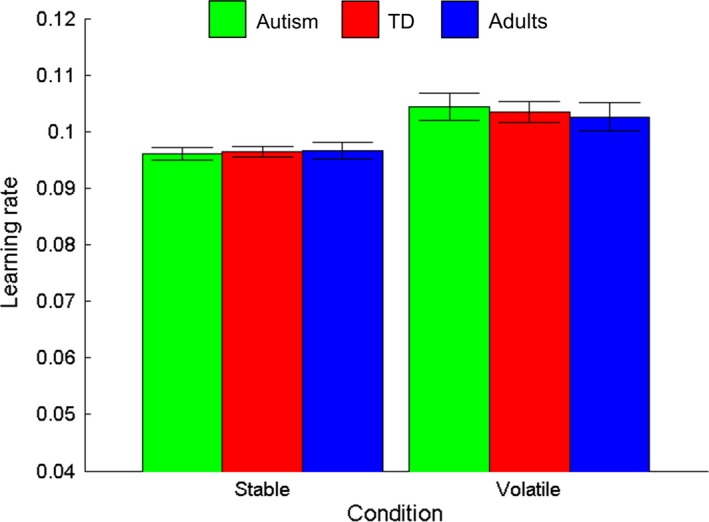
Learning rates in the stable and volatile conditions. Estimated learning rates in the stable and volatile conditions for autistic children (green), typically developing (TD) children (red) and typical adults (blue). Error bars represent ±1 standard error of the mean.

Next, we aimed to assess whether typically developing children and autistic children increased their learning rate as a function of volatility, in the same way as adults. Learning rates for the three groups are shown in Figure 6. As above, log‐transformed learning rates were used in the analysis. A mixed‐design ANOVA was conducted on learning rate estimates with condition (stable, volatile) as a within‐participants variable and group (children with autism, typical children, and typical adults) as a between‐participants variable. As expected, there was a main effect of condition, with higher learning rates in the volatile condition than the stable condition, *F*(1, 82) = 32.52, *p *<* *.001, ɳ_p_^2^ = .28. Unexpectedly, however, there was neither a significant effect of group, *F*(2, 82) = .02, *p *=* *.98, ɳ_p_^2^ < .001, nor a significant interaction between group and condition, *F*(2, 82) = .22, *p *=* *.81, ɳ_p_^2^ = .005. Thus, all groups increased their learning rate in the volatile condition to a similar extent. In order to judge the extent of a potential undetected effect, we compared the difference in log learning rates in the stable and volatile conditions between autistic and typical children, and found that the 95% confidence intervals of the group difference were tightly distributed around zero [−.03, .02]. Learning rates were not related to age, performance IQ or verbal IQ (all *p*s ≥ .39). Similarly, the difference in learning rates between the conditions was unrelated to age and ability (*p*s ≥ .58).

To investigate whether increased anxiety (as rated by parents) is related to reduced updating in the volatile condition (cf. Browning *et al*., 2015), we investigated the relationship between scores on the SCAS‐P and the difference in learning rates between the volatile and stable conditions for each participant. Parent ratings were not related to difference scores, *r *=* *.12, *p *=* *.35, nor to learning rates in either the stable or the volatile condition (*p*s ≥ .15).

**Figure 7:**
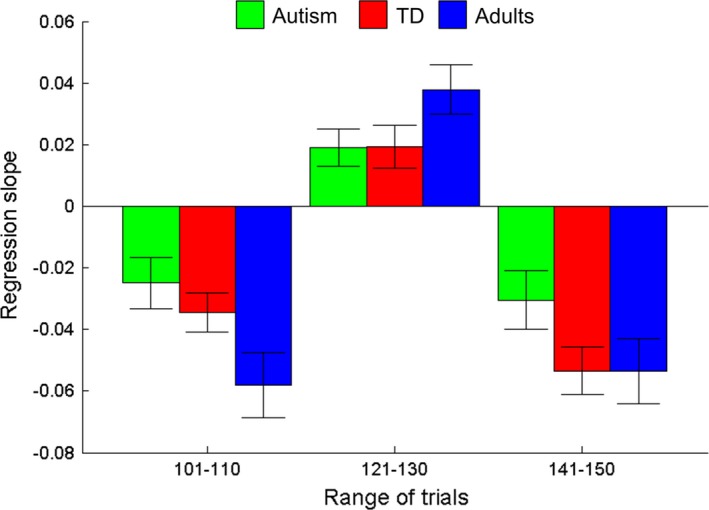
Gradients of regression lines fitted to participant choices after a switch in reward values. Mean regression slopes (±1 standard error of the mean) for the running average of four consecutive choices made by autistic children (green), typically developing (TD) children (red) and adults (blue) in the first 10 trials after the reward probabilities switched. For example, if the reward probability for the blue to the green chest switched from 80:20 to 20:80 at trial 100, participants became *less* likely to choose the blue chest (i.e. a negative slope). When the reward probability reversed again at trial 120, the participants became *more* likely to choose the blue chest (i.e. a positive slope).

In order to align our paradigm with previous studies of reversal learning in autism (cf. D'Cruz *et al*., 2013; Solomon *et al*., 2011; Robic *et al*., 2015), we investigated whether the autistic children could learn new reward probabilities as quickly as typically developing individuals. We calculated a running average of choices made by each participant over four consecutive trials, and fitted regression lines to the first ten running averages after the reward probabilities switched (i.e. trials 101–110, 121–130, and 141–150). The mean regression slopes for each group are shown in Figure 7.

We applied a square‐root transformation to the absolute slope coefficients in each condition in order to meet the normality assumption required for an ANOVA (Shapiro‐Wilk test, *p*s ≥ .06).^2^ Next, we conducted a mixed‐design ANOVA on the transformed absolute slope coefficients with trial interval (101–110, 121–130, 141–150) as the within‐participants factor, and group (autism, TD, adult) as the between‐participants factor. Mauchly's test demonstrated that sphericity could be assumed, χ^2^(2) = 3.91, *p *=* *.14. There was a significant within‐participants effect of trial interval on slope coefficients, *F*(2, 164) = 8.57, *p *<* *.001, ɳ_p_^2^ = .10.^3^ Repeated contrasts revealed that the slopes in the first interval (trials 101–110) were approximately as steep as slopes in the second interval (trials 121–130), *F*(1, 82) = 3.54, *p *=* *.06, ɳ_p_^2^ = .04, while slopes in the third interval (trials 141–150) were significantly steeper than those in the second interval, *F*(1, 82) = 16.16, *p *<* *.001, ɳ_p_^2^ = .17. Importantly, however, there was no overall group difference in slope coefficients, *F*(2, 82) = 2.82, *p *=* *.07, ɳ_p_^2^ = .06, nor an interaction between trial interval and group, *F*(4, 164) = .52, *p *=* *.72.

## Discussion

In this study, children with autism, typical children and typical adults completed a decision‐making task under two probabilistic reward schedules: a stable condition where the reward probabilities were fixed, and a volatile condition where the reward probabilities fluctuated. Based on recent Bayesian and predictive coding accounts of autism, we predicted that autistic children would assign more weighting to the outcome of recent trials than typical children and adults, and that they would not flexibly update their behaviour in response to volatility. In our task, these predictions would be manifest, respectively, as an increased learning rate compared to typical children, and a reduced tendency to increase their learning rate in the volatile condition. Contrary to these predictions, we found that children with autism had a similar learning rate to typical children and adults overall, and that they modified their learning rate to a similar extent as typical children and adults.

These results appear to be at odds with Bayesian and predictive coding accounts of autism. Children with autism employed the recent history of trial outcomes in a similar way to typical children, contrasting both Pellicano and Burr's (2012a) hypothesis of reduced use of priors in autism, and Van de Cruys *et al*.'s (2014) suggestion of highly precise prediction errors in autism, meaning that violations to expectations should be heavily weighted (see also Sinha *et al*., 2014). Furthermore, the children with autism were able to flexibly weight their prediction errors in order to update their learning rate in response to environmental volatility (cf. Van de Cruys *et al*., 2014). Why, then, do we find no differences in performance?

To date, Bayesian predictive models have arguably had most success in explaining how individuals with autism process sensory information. For example, these accounts have been linked to reports of reduced adaptation to high‐level sensory attributes, such as faces (Ewing, Pellicano & Rhodes, 2013; Fiorentini, Gray, Rhodes, Jeffery & Pellicano, 2012; Pellicano, Jeffery, Burr & Rhodes, 2007; Pellicano, Rhodes & Calder, 2013; Rutherford, Troubridg & Walsh, 2012; but see also Cook, Brewer, Shah & Bird, 2014) and numerosity (Turi, Burr, Igliozzi, Aagten‐Murphy, Muratori *et al*., 2015), as well as reduced use of contextual information in the rubber‐hand illusion (Palmer, Paton, Kirkovski, Enticott & Hohwy, 2015) and reduced filtering of signal‐from‐noise in motion displays (Manning, Tibber, Charman, Dakin & Pellicano, 2015; Zaidel, Goin‐Kochel & Angelaki, 2015). Thus, it is conceivable that atypical predictive mechanisms account for perception in autism, but may not extend to learning tasks, as in the current study. This proposal may not be too surprising, given that sensory symptoms are a core feature of the autistic phenotype (American Psychiatric Association, 2013), while general difficulties in learning have not been established (see Dawson, Mottron & Gernsbacher, 2008, for review). Indeed, previous research has suggested that individuals with autism learn reward probabilities as well as typical individuals in simple learning tasks (D'Cruz *et al*., 2013; Faja, Murias, Beauchaine & Dawson, 2013; Solomon *et al*., 2011), perform successfully in implicit learning tasks (Brown, Aczel, Jiménez, Kaufman & Plaisted‐Grant, 2010; Nemeth, Janacsek, Balogh, Londe, Mingesz *et al*., 2010; Foti, De Crescenzo, Vivanti, Menghini & Vicari, 2015), and even demonstrate enhanced visual statistical learning (Roser, Aslin, McKenzie, Zahra & Fiser, 2015).

Yet, differences between children with autism and typical children may become apparent using more complex learning tasks. For example, Pellicano, Smith, Cristino, Hood, Briscoe *et al*. (2011) showed that children with autism were slower than typical children to learn reward probabilities in a large‐scale foraging task, which required children to continuously update their spatial representations while remembering what locations they had already searched. Social situations may also pose particularly pronounced predictive processing challenges for autistic people (Gomot & Wicker, 2012; Lawson *et al*., 2014), as there is no simple one‐to‐one mapping between a cause and the sensory input. Thus, differences may well arise in a social version of our learning task (Behrens, Hunt, Woolrich & Rushworth, 2008). In such a task, autistic children may demonstrate difficulties in tracking the probability that a confederate would give the correct advice. Preliminary support for this suggestion comes from a recent study demonstrating reduced use of social information during a similar decision‐making task in typical people with high levels of autistic traits (Sevgi, Diaconescu, Tittgemeyer & Schilbach, 2016), although this will require replication in participants with a clinical diagnosis of autism. Predictive coding accounts have been proposed to explain a range of high‐level social abilities that may be affected in autism, such as theory of mind (Koster‐Hale & Saxe, 2013), interpersonal inference (Moutoussis, Fearon, El‐Deredy, Dolan & Friston, 2014) and interoception (Quattrocki & Friston, 2014; Seth, Suzuki & Critchley, 2011). Thus, further studies are required to probe the limits of atypical predictive processing in autism, and theoretical accounts will need to be updated to account for these. In particular, differences between children with autism and typical children may be manifest in more complex situations where the one‐to‐one mapping between events is even less clear, when higher levels of the predictive coding hierarchy are required (see also Qian & Lipkin, 2011). While our task was the ideal candidate for assessing decision‐making in an uncertain environment (Van de Cruys *et al*., 2014), it might not have been sufficiently challenging to reveal differences between children with and without autism.

A previous study with adults showed that anxious traits were related to reduced updating of behaviour in an aversive version of the task, in which incorrect responses were followed by electric shocks (Browning *et al*., 2015). In this study, we did not find a relationship between levels of parent‐reported trait anxiety and task performance. Yet, the children with autism were reported by their parents to have elevated levels of anxiety overall. Thus, even those children with high levels of anxiety were able to update their behaviour to environmental volatility in a typical fashion. The lack of the predicted relationship may have been because we did not use an aversive reinforcer in this task, unlike Browning *et al*. Future research could investigate relationships between task performance and child‐reported anxiety, or measures of state anxiety (e.g. heart rate, saliva cortisol levels).

As well as addressing specific proposals from Bayesian predictive coding models, our results add more generally to the reward learning literature in autism. Previous studies have revealed subtle differences in reversal learning between individuals with and without autism. For example, D'Cruz *et al*. (2013) reported that individuals with autism needed more trials to achieve a criterion of 8 correct out of 10 consecutive responses after a switch, and were more likely to return to the previously reinforced response after having selected the new correct choice. Robic *et al*. (2015) reported that fewer autistic individuals reached a criterion of 60% correct than typical individuals when reward probabilities fluctuated, and that autistic individuals were more likely to maintain their response after a failure than typical individuals. Furthermore, Solomon *et al*. (2011) reported that individuals with autism were less likely to maintain their response after a success, and Solomon, Frank, Ragland, Smith, Niendam *et al*. (2015) reported selective difficulties with learning high‐probability pairings but not lower‐probability pairings. The participants involved in the current study differed substantially from those tested by Robic *et al*. (2015) and Solomon *et al*. (2011, 2015), who tested adult participants, and D'Cruz *et al*. (2013), who tested participants of a wide age range (between 8 and 44 years). However, it is worth noting that the current paradigm is not directly comparable to these previous studies which did not manipulate the reward value. It does not make sense to assess the proportion correct in this study in the same way as in these previous studies, as on a given trial, the optimal choice may not have been the same as the most frequently rewarded choice. However, the children with autism appeared to change their behaviour just as quickly as typical children and adults after a switch in the current experiment.

The discrepancy between the current study and previous studies of reversal learning raise the intriguing possibility that a certain amount of environmental volatility may actually help children to adapt to new situations. Previous studies of reversal learning in autism have been unable to dissociate whether difficulties changing responses after a switch result from slower switching per se, or from individuals with autism building a more stable state prior to the switch. When there is no reward value information, the optimal strategy is to consistently choose the option with the highest reward probability. Under these conditions, individuals with autism may reach a very stable state, which means that they will need more evidence to reject their current model and shift to a new model. Yet, the addition of randomly fluctuating reward value information, as in our paradigm, may prevent children with autism getting into such a stable initial state. Thus, it is possible that reward value fluctuations in the stable condition help children with autism to deal with the bigger change that occurs when they move into the volatile phase of the experiment. While speculative, this may suggest that some divergence from routines may be beneficial to autistic children in adapting to everyday situations. To investigate this possibility further, it will be important to compare different learning paradigms in the same samples of participants.

In sum, our study shows that children with and without autism can learn about the statistics of the reward environment in a similar way to typical adults, by updating their learning rate when the environment becomes volatile. The typical performance of children with autism contrasts hypotheses emerging from recent Bayesian and predictive coding accounts of autism. While we believe that there is much mileage in these approaches, they need to be explicitly laid out in order to make clear predictions from behavioural studies. Van de Cruys *et al*. (2014) made one such prediction: ‘We predict that things will go awry in ASD [autism spectrum disorder] when the probabilistic structure changes during the experiment, for instance when the predictability of a cue changes across blocks’ (p. 655). Our results do not support this prediction and argue against the suggestion that precision is set uniformly high in individuals with autism. Bayesian and predictive coding accounts may be best suited to explaining atypical sensation and perception in autism, and may not generalize to learning tasks. Yet, future research is needed in order to determine whether atypical predictive processing may be apparent for more complex learning tasks, such as those requiring individuals with autism to learn about social aspects of the environment. Further insights into learning mechanisms in autism may be gleaned by using implicit measures (e.g. eyetracking or neuroimaging) in conjunction with our behavioural task.

## Notes

1 The term ‘autistic person’ is the preferred language of many people on the spectrum (see Sinclair, 1999; Kenny, Hattersley, Molins, Buckley, Povey *et al*., 2016). In this article, we use this term as well as person‐first language to respect the wishes of all individuals on the spectrum.

2 Note that the same pattern of results was obtained without transformation.

3 Note that this effect remained significant (*p *<* *.001) after applying a Greenhouse‐Geisser correction.
